# Raw or Cooked? Exploring Vegetable Acceptance Among Chilean Children from Different Socioeconomic Backgrounds

**DOI:** 10.3390/foods14071133

**Published:** 2025-03-25

**Authors:** Karinna Estay, Victor Escalona

**Affiliations:** 1 Departamento de Agroindustria y Enología, Facultad de Ciencias Agronómicas, Universidad de Chile, Av. Santa Rosa 11315, La Pintana 8820808, Chile; 2 Departamento de Producción Agrícola, Facultad de Ciencias Agronómicas, Universidad de Chile, Av. Santa Rosa 11315, La Pintana 8820808, Chile

**Keywords:** food acceptability, socioeconomic status, vegetable liking, preparation methods, children’s nutrition

## Abstract

This study explores how socioeconomic status (SES) influences the acceptability of familiar vegetables in Chilean children aged 9–10, examining its relationship with sex, BMI, and preparation methods. A sensory evaluation was conducted in two stages to assess responses across sensory dimensions: appearance, aroma, taste, texture, and overall-opinion. In the first stage, 363 children evaluated eight vegetables: tomatoes, lettuce, corn, cucumber, carrots, beets, broccoli, and cauliflower. Results show significant differences across samples for all sensory dimensions (*p* < 0.0001), with tomatoes, lettuce, corn and cucumber receiving the highest ratings. The second stage involved 191 children, who evaluated the three least preferred vegetables (carrots, beets, and cauliflower) in raw and cooked forms. Raw vegetables received higher ratings than cooked across all sensory dimensions, except for beets in appearance, where raw scored lower. Significant differences were found for carrots and cauliflower (*p* < 0.0001), while the difference for beets was not statistically significant. SES had limited influence on vegetable liking, while BMI showed some interactions with texture and aroma ratings. These findings suggest lower vegetable consumption in vulnerable groups may not stem from reduced liking of familiar vegetables. Increasing availability could help, along with exploring preparation methods for less-liked vegetables, particularly raw options, which appear promising.

## 1. Introduction

Promoting vegetable consumption among children is a crucial public health priority due to its significant role in preventing chronic diseases and fostering overall health [1]. Diets rich in vegetables are associated with a reduced risk of non-communicable diseases, including obesity, type 2 diabetes, and various types of cancers [2,3,4]. Additionally, observational studies indicate that high fruit and vegetable intake is linked to improved psychological well-being [5]. Despite these recognized health benefits, vegetable intake among children remains alarmingly low. Studies indicate that many children globally fail to meet the recommended intake of at least 400 g per day [6,7,8]. In the United States, for example, fewer than 10% of children meet this recommendation [8], and only 2% of adolescents achieve this target [9]. Similarly, in Europe, only 22.6% of children aged 6 to 9 consume vegetables daily [10]. A similar pattern is observed in Chile, where the latest National Food Consumption Survey reveals that children aged 6–13 years consume an average of just 167 g of vegetables per day, with intake decreasing in lower socioeconomic groups [11].

Socioeconomic status (SES), which encompasses disparities in income, education, and access to resources, plays a crucial role in shaping dietary habits and may influence vegetable acceptance in childhood [12]. These dietary disparities are also reflected in differences in body mass index (BMI), with higher rates of overweightness and obesity observed among children from lower socioeconomic backgrounds [13]. Specifically, among Chilean children aged 6 to 12 years, overweightness and obesity rates are 20.5% higher in vulnerable groups compared to more affluent ones (19.6% vs. 41% in first grade and 23.1% vs. 41.6% in fifth grade) [14].

Numerous studies have shown that vegetables are among the foods liked least by children [15]. Their acceptance, however, can improve through repetitive exposure and positive experiences, as familiarity reduces rejection [16,17]. Nevertheless, not all familiar vegetables are equally well accepted [16]. Sensory factors, such as taste, texture, and visual appeal, play a crucial role in their acceptability [18,19]. For example, dark leafy greens are often rejected due to their bitterness, while cruciferous vegetables pose challenges because of their strong aroma [20]. Additionally, the low energy content of vegetables makes them less appealing to children, who naturally prefer sweeter and more energy-dense foods [19].

While existing research suggests that higher-income groups tend to consume more vegetables than lower-income groups [12], it remains unclear whether this disparity is driven by differences in taste preferences. Since food consumption, particularly in children, is closely tied to taste preferences [16], it is essential to determine whether children from lower socioeconomic backgrounds have a lower liking for familiar vegetables compared to their higher-income peers. This distinction is crucial, as it can reveal whether improving accessibility alone would be sufficient to increase consumption or if additional barriers, such as lower acceptability, must also be addressed.

Chile provides a particularly relevant context for this investigation, as its significant economic inequality [21], combined with high rates of childhood overweightness and obesity, highlights the need to examine how socioeconomic disparities shape dietary habits and health outcomes.

This study aimed to examine children’s vegetable preferences in relation to socioeconomic factors and sensory attributes, specifically assessing the acceptance of eight commonly consumed vegetables in familiar preparation forms in Chile. Additionally, the impact of preparation methods on the least-liked samples was evaluated by comparing raw and cooked versions to identify potential strategies for enhancing acceptance. By considering variables such as socioeconomic status, sex, and BMI, this research seeks to provide insights that can help improve vegetable acceptance among children across diverse socioeconomic backgrounds.

## 2. Materials and Methods

### 2.1. Participants

A total of 554 children, aged 9 to 10 years, participated in the study, evenly distributed between boys and girls. The age range of 9 to 10 years was chosen to ensure a homogeneous group with sufficient reading and comprehension skills to independently understand the sensory evaluations and complete the questionnaires [22,23]. Participants were recruited from schools located in the metropolitan urban area of Santiago, Chile. A total of 16 schools were involved in the study. Fifteen were selected for data collection (three per socioeconomic group: low, low-medium, medium, medium-high, and high), while an additional school from the low socioeconomic group was used exclusively for a pilot test. The classification of schools by socioeconomic status (SES) was based on the methodology of the System for Measurement of the Quality of Education (SIMCE), a national instrument administered by the Ministry of Education in Chile. SIMCE uses cluster analysis to group schools into five SES categories, considering variables such as parents’ educational attainment, household income, and the School Vulnerability Index (IVE). The IVE assesses factors such as poverty, unemployment, access to basic services, availability of community resources, and school infrastructure and climate, with a scale ranging from 0 (no detected vulnerability) to 100 (maximum vulnerability). For this study, schools (based on the categorization of fourth-grade students) were classified into five socioeconomic status (SES) groups. For low SES, parents had an average of 8 years of education (equivalent to completing middle school, the same for mothers and fathers), with an IVE of more than 81%; for low-medium SES, parents had an average of 10 years of education, with an IVE between 61.01% and 81%; for medium SES, parents had an average of 12 years of education (equivalent to completing high school, the same for mothers and fathers), with an IVE between 37.01% and 61%; for medium-high SES, parents had an average of 14 years of education (also equivalent to completing high school, the same for mothers and fathers), with an IVE between 5.01% and 37%; and for high SES, parents had an average of 16.5 years of education (mothers: 16 years, fathers: 17 years; equivalent to a technical degree and a university degree, respectively), with an IVE of less than 5% [24]. Children were eligible to participate if they were part of the selected fourth-grade classes, had no known allergies to fruits or vegetables, and provided informed consent signed by their parents or guardians, as well as their own written assent. In the second stage, participants were selected from parallel fourth-grade classes within the same schools to ensure no child participated in both stages. 

### 2.2. Research Stages

**Figure 1:**
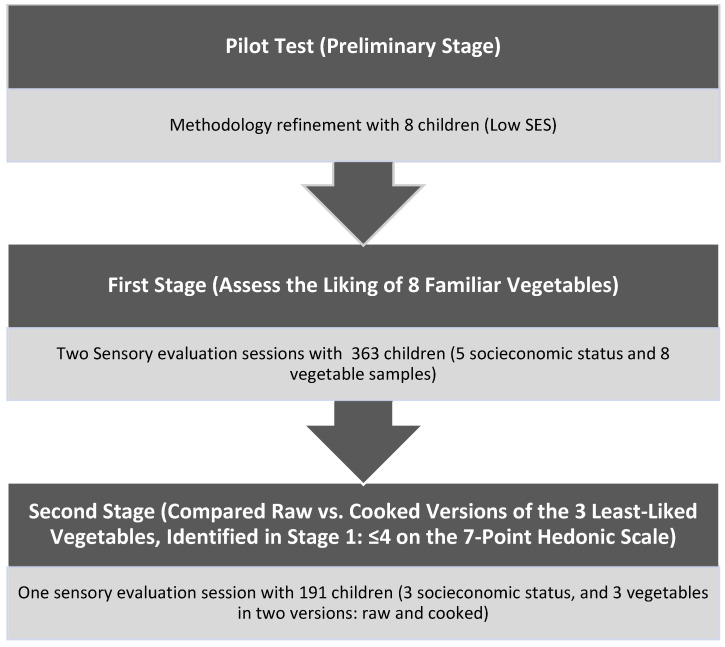
Study design flowchart.

To structure the study and systematically assess children’s vegetable acceptance, the research was divided into three stages: pilot test, first stage and second stage (Figure 1).

Pilot Test: Before starting the first stage, a pilot test was conducted with eight fourth-grade participants from a low-SES school. This preliminary assessment refined the tasting procedures and ensured that both the sensory test and questionnaires were age-appropriate. While no reportable data were collected, the pilot test was critical in defining the research methodology.

First Stage: Evaluating Familiar Vegetables: The first stage involved 363 children from schools representing five SES levels—low, low-medium, medium, medium-high, and high. Participants evaluated eight vegetable samples over two tasting sessions, with a pause between sessions. The samples were selected for their familiarity/commonness to children in Santiago. The selection of eight vegetable samples was based on their availability and relevance within the Chilean market. Data from the main wholesale markets supplying Santiago were analyzed, focusing on the 20 vegetables with the highest volumes, according to the Chilean Agricultural Policy and Research Office [25]. This list was cross-referenced with information from the National Food Consumption Survey (ENCA) [11] (which reported the three most-consumed vegetables which were included in the selection), seasonal availability during autumn—the data collection period—and the exclusion of vegetables commonly used as condiments (e.g., chili peppers, garlic, and cilantro). The resulting list included tomatoes, lettuce, corn, cucumber, carrots, beets, broccoli, and cauliflower. To ensure consistency in the quality and characteristics of the vegetable samples, all vegetables were purchased from the same supermarket throughout the study. This approach aimed to minimize variability in factors such as freshness, size, and origin, ensuring that all participants evaluated samples with comparable attributes. Additionally, all vegetables were selected following standardized criteria for ripeness and appearance to maintain uniformity across testing sessions. To ensure familiarity, this selection was validated with school cafeterias representing low, medium, and high socioeconomic groups. Preparation methods for the samples in their familiar format were defined by the research team in collaboration with school cafeteria staff. Table 1 presents the eight selected vegetables and their respective preparation and serving methods.

To ensure uniformity in the sensory evaluation, the preparation of the eight samples was standardized in terms of cutting method, dressing quantities, and cooking time, aiming to maintain consistent presentation, texture, and flavor. Tomatoes (globe fresh tomato) were peeled, cut into six equal wedges, and seasoned just before serving with a 1:3:10 (salt/oil/water) dressing, using 3 g of dressing per 5 g of tomato. Lettuce (iceberg lettuce) was sliced into thin strips (approximately 0.5 cm wide and 3 cm long) and seasoned with a 1:5:10 (salt/oil/lemon juice) dressing, applying 5 g of dressing per 10 g of lettuce. Cucumbers (Straight Eight cucumber) were peeled and cut into small pieces (approximately 0.5 cm thick) and dressed with the same 1:5:10 (salt/oil/lemon juice) dressing, using 5 g of dressing per 20 g of cucumber. Carrots (Danvers type) were peeled, boiled in 2 L of water with 3 tablespoons of salt for 5 min after reaching a rolling boil, and then sliced into 0.5 cm thick rounds. Corn kernels (frozen sweet maize) were boiled in 2 L of salted water (3 tablespoons of salt) for 8 min, then drained. Broccoli (head broccoli) and cauliflower (white cauliflower) were cut into small florets (approximately 1 cm), boiled in 2 L of salted water (3 tablespoons of salt) for 4 min, and then drained. Carrots, corn, broccoli, and cauliflower were not additionally seasoned. Beets (red beet) were boiled whole for 50 min, transferred to cold water for 10 min, and then peeled, halved lengthwise, and sliced into 0.5 cm thick pieces. Beets were seasoned with the same 1:5:10 (salt/oil/lemon juice) dressing, using 5 g of dressing per 10 g of beets.

Each tasting session took place in collective rooms within each school, which were spacious enough to accommodate a full class of 36–40 students. However, to ensure a controlled environment, groups of 8 to 12 children participated in each session. Each child was seated individually with space between them to minimize peer interaction and reduce the potential for social bias. All sensory evaluations were conducted at the same time across all schools, specifically after the second recess, which occurred at midday. All vegetable samples were prepared fresh on the same morning, with warm samples briefly reheated before serving to maintain consistency and salad samples seasoned just minutes before serving.

The sessions were led by the principal investigator, two research assistants, and a schoolteacher, ensuring a team structure that provided a ratio of one adult for every three children, or one researcher for every four children. At the beginning of each session, the research team provided clear instructions, explaining the correct procedures for the evaluation and guiding the children through the questionnaire. Emphasis was placed on ensuring that each child evaluated the samples individually. Each child independently completed the questionnaire on tablets via Qualtrics XM, ensuring data completeness with mandatory responses. The vegetable samples were presented in individual containers with lids, following a counterbalanced serial monadic sequence, with the order determined by a Williams Latin square design [26]. To enhance engagement, the evaluation was framed as a discovery game, with children unveiling one sample at a time.

To assess the liking of the samples, children used a 7-point hedonic facial scale (with numbers, words, and faces), which is appropriate for their age group [23]. Liking was measured across five sensory dimensions: appearance, aroma, taste, texture, and overall opinion. Children were instructed to first evaluate the appearance, followed by the aroma, and finally taste, texture, and overall liking.

The children’s weight and height were measured using a digital scale (Tanita BF-689) and a mechanical stadiometer (SECA 213). Body mass index (BMI) was calculated as weight (kg) divided by height squared (m^2^), and BMI categories were determined using Chile’s gender-specific age percentile growth tables [27]. The demographic characteristics of the participants in this stage are summarized in Table 2.

**Figure 2:**
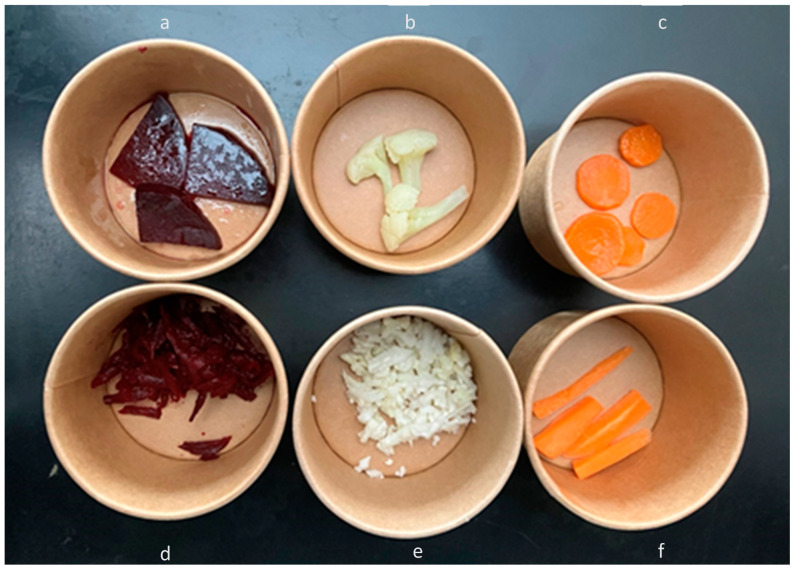
Least-liked familiar vegetables in their raw and cooked versions. Labeled from (**a**–**f**), in order from top to bottom and left to right: (**a**) cooked beet, (**b**) cooked cauliflower, (**c**) cooked carrot, (**d**) raw beet, (**e**) raw cauliflower, and (**f**) raw carrot.

Second Stage: Exploring Alternative Preparations: Building on the findings from the first stage, the second stage targeted the three vegetables with the lowest mean overall liking scores (≤ 4 on the 7-point scale): carrots, cauliflower, and beets. A new cohort of 191 children from the same schools (but different classes) representing three SES levels (low, medium, and high) participated. This stage aimed to explore whether alternative preparation methods—raw and cooked—could enhance the acceptance of these vegetables. In this single tasting session, each vegetable was evaluated in both preparations, as shown in Table 3 and Figure 2.

To standardize the sensory evaluation, the raw samples were prepared as detailed below. Beets (red beet) were peeled, grated raw, and seasoned with a 1:10:5 (salt/oil/lemon juice) dressing, applying 5 mL of dressing per 250 g of grated beets. Cauliflower (white cauliflower) was grated raw and seasoned with the same 1:10:5 (salt/oil/lemon juice) dressing, using 5 mL of dressing per 250 g of grated cauliflower. Carrots (Danvers type) were peeled and cut into sticks of approximately 4 to 5 cm in length. No additional seasoning was applied. The preparation of cooked carrots, cooked beets, and cooked cauliflower remained the same as described in the first stage.

The setup for this stage mirrored that of the first, maintaining the same team structure and methodology for presenting and evaluating the samples. Participants rated the vegetables using the same 7-point hedonic facial scale, assessing the same five sensory dimensions. The demographic and BMI characteristics of this cohort are presented in Table 4.

### 2.3. Data Analysis

All statistical analyses were conducted using R Studio (version 2024.04.2, build 764). A significance level (α) of 1% was applied to ensure robust results and minimize the likelihood of Type I errors, particularly given the exploratory nature of the study and the multiple comparisons performed. Fisher’s least significant difference (LSD) test was used for post-hoc comparisons in all ANOVA models.

#### 2.3.1. Analysis of Familiar Vegetable Liking

To assess how sociodemographic and anthropometric factors influenced children’s liking of familiar vegetable samples, a 4-way ANOVA model was performed. The factors included socioeconomic status (SES) (high, medium-high, medium, medium-low, and low), child sex (female and male), BMI categories (normal weight and overweight–obese), and samples (tomatoes, lettuce, corn, cucumber, carrots, beets, broccoli, and cauliflower). Initially, all interaction terms were included in the model, but only statistically significant interactions were retained in the final version. Separate analyses were conducted for each sensory dimension: appearance, aroma, taste, texture, and overall opinion. This analysis included participants from the first stage (*n* = 363).

#### 2.3.2. Analysis of Liking for Raw vs. Cooked Samples

To explore the influence of preparation method (raw vs. cooked) on children’s liking of non-preferred vegetable samples, a second 4-way ANOVA model was conducted. The factors considered were socioeconomic status (SES) (high, medium, and low), child sex (female and male), BMI categories (normal weight and overweight–obese), and samples (cooked beet, cooked cauliflower, cooked carrot, raw beet, raw cauliflower, and raw carrot). As with the previous model, all interaction terms were initially included and then reduced to significant ones. This model was applied separately to each sensory dimension: appearance, aroma, taste, texture, and overall opinion. The analysis included participants from the second stage (*n* = 191).

## 3. Results

### 3.1. Factors Influencing Liking of Familiar Vegetables

**Figure 3:**
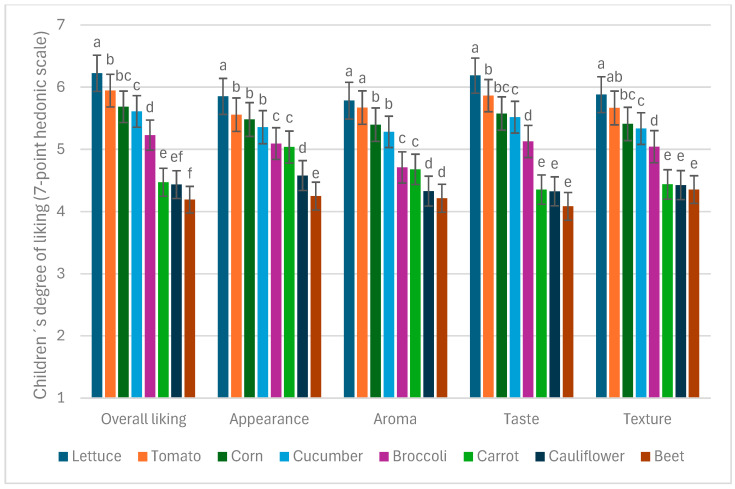
Liking of familiar vegetable samples across sensory dimensions. Mean ratings on the 7-point hedonic scale and standard error of the mean for children (*n* = 363) for familiar vegetable liking across sensory dimensions (appearance, aroma, taste, texture, and overall opinion). Within each sensory dimension, means with different superscripts are significantly different at *p* < 0.01.

**Figure 4:**
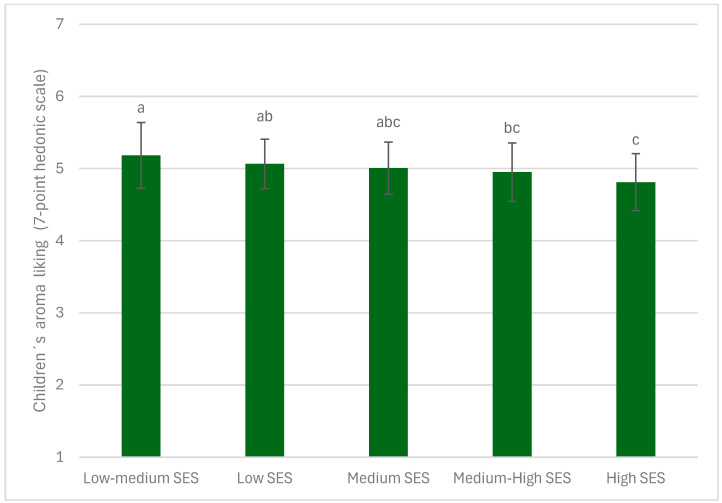
Association between socioeconomic status and the liking of vegetable aroma. Mean ratings on the 7-point hedonic scale and standard error of the mean for children (*n* = 363) regarding the liking of familiar vegetable appearance. Means with different superscripts are significantly different at *p* < 0.01.

When analyzing the effect of children’s SES, sex, BMI, and vegetable samples on their liking of familiar vegetables for each sensory dimension separately, no significant differences were found based on the child’s sex or BMI. However, significant differences were consistently observed across all sensory dimensions (appearance, aroma, taste, texture, and overall opinion) between the different vegetable samples evaluated (*p* < 0.0001, F = 58.66 for overall liking, F = 35.61 for appearance, F = 43.99 for aroma, F = 57.07 for taste, and F = 41.80 for texture). Specifically, lettuce, tomato, corn, and cucumber were the most liked samples, while carrot, cauliflower, and beets were the least liked (Figure 3). SES showed significant differences only in the evaluation of the sensory dimension of aroma (*p* < 0.01, F = 3.82). In particular, children from high socioeconomic statuses rated the aroma of vegetables lower compared to those from low and low-medium SES, as shown in Figure 4.

**Figure 5:**
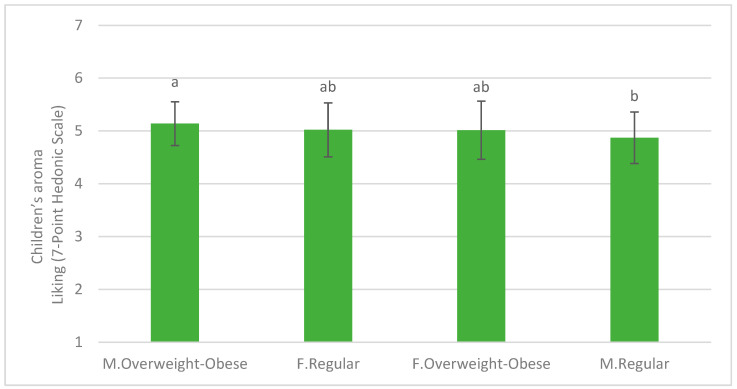
Interaction between BMI and sex on the liking of vegetable aroma. Mean ratings on the 7-point hedonic scale and standard error of the mean for children (*n* = 363) regarding the liking of familiar vegetable aroma. Means with different superscripts are significantly different at *p* < 0.01.

With respect to the interaction between BMI and sex on the degree of liking, significant differences were found in the evaluation of the aroma dimension (*p* < 0.001, F = 8.36). These differences were found only in males, where overweight–obese males rated the samples higher than normal weight males for aroma (Figure 5).

### 3.2. Impact of Preparation Method on Liking of Non-Preferred Vegetables

**Figure 6:**
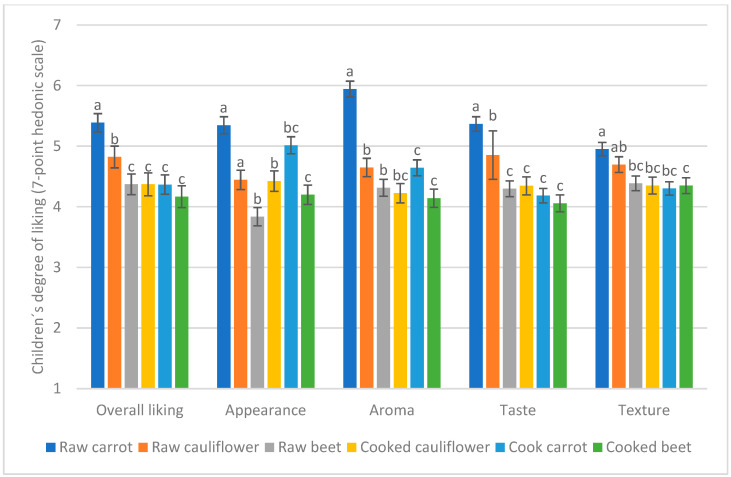
Differences in liking between raw and cooked non-preferred vegetables across sensory dimensions. Mean ratings on the 7-point hedonic scale and standard error of the mean for children (*n* = 191) across sensory dimensions (appearance, aroma, taste, texture, and overall opinion). Within each sensory dimension, means with different superscripts are significantly different at *p* < 0.01.

When analyzing the impact of the preparation method on the degree of liking of non-preferred vegetables, no significant differences were found based on the child’s SES, sex, or BMI. However, significant differences were consistently observed across all sensory dimensions (appearance, aroma, taste, texture, and overall opinion) between the different vegetable samples (*p* < 0.0001, F = 8.99 for overall liking, F = 17.19 for appearance, F = 7.49 for aroma, F = 10.70 for taste, and F = 3.53 for texture). Specifically, raw carrot was liked significantly more than cooked carrot, and raw cauliflower was liked significantly more than cooked cauliflower. No significant differences were found for beets in their raw versus cooked preparation across all sensory dimensions evaluated (Figure 6).

Regarding the interaction between SES and BMI on the degree of liking, significant differences were observed in the texture dimension evaluation (*p* < 0.01, F = 4.95). These differences were found only in the high SES group, where overweight–obese children gave higher hedonic scores compared to children with normal weight.

## 4. Discussion

### 4.1. Liking of Familiar Vegetables Across Socioeconomic Groups

This study evaluated the liking of eight familiar vegetables among children from different socioeconomic backgrounds. The results show no significant differences in overall vegetable liking across socioeconomic (SES) groups. Previous research has established a correlation between children’s vegetable liking and their consumption patterns [28,29], with higher liking scores being associated with greater intake [30]. Although this study did not directly measure vegetable consumption, national dietary surveys indicate that children from lower SES groups consume fewer vegetables than those from higher SES groups [11]. One possible explanation for this discrepancy is food neophobia (FN). A previous study [31] found that SES influences children’s willingness to try unfamiliar vegetables, with lower-SES children exhibiting higher FN levels. Since the vegetables tested in this study were familiar, the effect of FN observed in previous studies—where SES influenced vegetable acceptance by affecting willingness to try unfamiliar vegetables—should not apply in this case due to their familiar nature. This could explain why no SES-related differences were observed in overall liking.

Although SES did not significantly impact overall liking—nor the dimensions of taste, texture, or aroma—it did influence appearance ratings. Specifically, children from higher SES groups rated the appearance of vegetables lower, and a trend was observed where aroma ratings decreased as SES increased. However, these stricter evaluations in appearance and aroma did not translate into lower overall acceptance. Interestingly, this pattern aligns with previous findings on novel vegetable acceptance, where children from higher SES backgrounds were more willing to try unfamiliar vegetables [31]. This could be attributed to greater exposure to a wider variety of food in children from high SES, making them more open to new experiences but also more critical in their evaluations, particularly regarding visual aspects. Nonetheless, the fact that lower appearance ratings did not negatively affect taste, texture, or overall liking suggests that while higher-SES children may be stricter in their initial sensory assessments, this does not necessarily translate into reduced acceptance of familiar vegetables.

### 4.2. Liking of Different Vegetable Types

Among the eight familiar vegetables evaluated, the most accepted were lettuce, tomato, corn, and cucumber. This aligns with the latest National Food Consumption Survey [11], which also identifies tomato, lettuce, and carrots as the three most-consumed vegetables in Chile. The consistency between high acceptance and high consumption reinforces the idea that repeated exposure plays a crucial role in shaping children’s preferences [32]. However, despite being the third most-consumed vegetable in Chile, carrot was not among the most preferred in our study. This may be explained by its preparation method: in the first research stage, carrot was presented cooked, reflecting how it is commonly served in school cafeterias (see Materials and Methods). However, in its raw form—measured in the second research stage—carrot achieved similar acceptance levels to corn, suggesting that familiarity alone does not determine preference, but rather the combination of exposure and preparation method. The high acceptance of cucumber is consistent with previous studies in different countries, highlighting its widespread appeal among children [33].

Following these four highly accepted vegetables, broccoli was the next most liked. Broccoli is often portrayed in media as an undesirable vegetable among children, reinforcing its negative reputation. For example, in the movie *Inside Out* (Pixar, 2015), broccoli is depicted as a food that children instinctively reject, reflecting a common stereotype in Western media. However, findings from the present study indicate that children rated broccoli favorably, aligning with previous cross-cultural studies where children from Chile, China, and the United States also evaluated it positively, scoring 5 or above on a 7-point hedonic scale [33]. In the present study, although its aroma received slightly lower scores (4.7), its appearance, taste, and texture were rated more favorably, contributing to its high overall acceptance. These results suggest that, despite negative cultural associations, broccoli may have a higher intrinsic acceptability than often assumed.

The least accepted vegetables were carrot, cauliflower, and beet, all evaluated in their cooked versions, with average scores between 4 and 4.5 across all sensory dimensions—placing them within the neutral evaluation range—except for the appearance of cooked carrot, which received a higher score of 5, placing it in the acceptability range. Unlike cauliflower and beet, which are mostly consumed cooked in Chile, carrot is commonly eaten both raw and cooked. It is possible that children evaluated its cooked appearance based on an expectation of its raw form (see Section 4.3 for further details).

Notably, none of the eight evaluated vegetables received mean scores below 4, which represents a neutral evaluation, and five were rated in the acceptability range (5 or above), reinforcing their potential to increase familiar vegetable consumption among Chilean children across all socioeconomic (SES) groups. This is particularly relevant for children from lower SES backgrounds, given their more restricted vegetable consumption and their higher BMI [34]. These findings suggest that low vegetable consumption in low SES groups may not result from an inherent dislike but rather from other barriers such as availability, exposure, or preparation methods. This highlights the importance of strategies that go beyond simple exposure and consider how vegetables are presented and integrated into children’s diets, particularly in school settings where many dietary habits are formed.

### 4.3. Effect of Preparation Method on Acceptance of Least-Liked Vegetables

The second research stage focused on the three least-liked familiar vegetables from the first stage—carrot, cauliflower, and beet—by testing their acceptance in both raw and cooked forms. Results show that children generally preferred these vegetables when served raw, regardless of socioeconomic status. Although raw beets received higher mean scores than cooked beets in overall liking, this difference was not statistically significant. However, raw beets consistently scored higher across all sensory dimensions except appearance, where they were rated significantly lower than cooked beets, marking the only evaluation within the “dislike” range. This discrepancy may be attributed to the low familiarity with raw beet in Chilean cuisine, considering that familiarity plays a key role in food acceptance during childhood [35]. Nonetheless, despite the initial lower scores in appearance, raw beet was well rated in terms of taste and texture, leading to a higher overall liking than expected. Notably, despite its sweet taste, a well-established factor enhancing food acceptability in childhood [36], beets remained the least-liked vegetable in both preparations. A similar pattern was observed for cauliflower, with the cooked version receiving higher ratings in appearance, likely due to its greater familiarity, as it is predominantly consumed cooked in Chile. However, unlike beet, raw cauliflower achieved high levels of acceptability after being tasted by children, particularly in overall liking. This suggests that raw cauliflower could serve as a viable alternative to increase vegetable consumption among children. Raw carrot, on the other hand, was rated higher than cooked carrot across all sensory dimensions, reinforcing its greater acceptance in its raw form. Unlike beet and cauliflower, carrot is commonly consumed both raw and cooked in Chile, and yet in this study, a clear preference for the raw version was observed. Given its high acceptance and ease of preparation, raw carrot could be a practical option to encourage vegetable consumption among school-aged children.

These findings underscore the crucial role of food preparation methods in shaping children’s vegetable preferences. Previous research has shown that repeated exposure combined with favorable preparation methods can significantly enhance vegetable acceptance [16]. Presenting vegetables in different forms has also been shown to influence children’s willingness to consume them [29,37]. The positive response to raw vegetables is particularly relevant, as raw preparations require less effort and time—factors mothers have identified as key barriers to vegetable consumption in children [38]. Encouraging raw vegetable intake could thus serve as a practical and effective strategy to improve children’s overall vegetable consumption while addressing logistical challenges in food preparation.

### 4.4. Influence of Sex and BMI on Vegetable Liking

Sex and BMI, when analyzed independently, did not significantly affect vegetable liking. However, a significant interaction was found in the aroma evaluation of familiar vegetables among males, with overweight–obese males rating the samples higher than their normal-weight counterparts. The relationship between BMI and olfactory sensitivity is complex and not widely studied. Skrandies and Zschieschang (2015) observed reduced olfactory sensitivity in individuals with higher BMI compared to those with normal weight [39], which could partly explain the greater acceptability of the less-preferred samples analyzed. Similarly, some authors have hypothesized that women have an anatomical advantage that may manifest as greater olfactory perception [40]. Notably, two of the six samples evaluated in this second research stage belonged to the Brassica family (cauliflower, both raw and cooked), known for their strong sulfur aromas, which often impact acceptability. In this context, lower perception could lead to higher acceptance. However, this remains a hypothesis that requires further validation.

Previous research suggests that aroma and appearance are the sensory dimensions least correlated with overall vegetable liking in children [33]. The present findings align with this, as differences in aroma ratings by sex and BMI did not translate into variations in overall liking. While some studies report that girls tend to prefer vegetables more than boys [41,42], the overall effect of sex on vegetable liking remains inconsistent.

Similarly, BMI did not significantly influence vegetable liking, which aligns with findings from previous research [31]. This lack of association may be explained by the fact that overweightness and obesity result from an imbalance between energy intake and expenditure [43], with studies indicating that higher BMI in children is often associated with greater consumption of ultra-processed foods rather than lower vegetable preference [44]. However, other studies have found that high BMI in children is linked to a limited intake of vegetables [45]. In this regard, evidence suggests that the relationship between SES, vegetable consumption, and ultra-processed food intake is complex. For example, Cooke et al. (2006) observed that children from affluent families who consumed fewer vegetables did not compensate by increasing their intake of ultra-processed foods [46]. In contrast, other studies found that younger children from low-SES backgrounds not only consumed fewer vegetables but also exhibited higher consumption of ultra-processed foods [47], exacerbating their nutritional vulnerability.

Additionally, an interaction between SES and BMI was observed for the texture evaluation of the least-liked vegetables. Notably, significant differences emerged only in the high-SES group, where overweight–obese children rated texture more favorably than those with normal weight. The mechanisms behind this effect were not analyzed in the present study. Further research is needed to better understand whether differences in dietary habits or exposure to food textures among children with different BMI levels may influence their evaluation of the degree of liking for the texture of vegetables.

This study provides valuable insights into socioeconomic differences in vegetable acceptance by including children from the full range of socioeconomic backgrounds in Chile, from the most vulnerable groups to the highest SES levels. Populations with a low socioeconomic position are often underrepresented in research, making this study particularly relevant for understanding disparities in dietary habits. Additionally, vegetable liking was assessed using real food samples, with children’s direct hedonic responses providing an ecologically valid measure of acceptance. A potential limitation of this study is that SES was classified at the school level rather than individually for each student. While this approach may not fully capture household-level socioeconomic variability, it is based on a national classification system (SIMCE) administered by the Chilean Ministry of Education, specifically designed for fourth-grade students—the target group of this study. This ensures a standardized and reliable estimate of SES differences across schools. However, the lack of individual SES data should be considered when interpreting the results.

## 5. Conclusions

The findings of this study indicate that the liking of familiar vegetables does not differ significantly among children from different socioeconomic backgrounds. This suggests that the lower vegetable consumption reported in lower socioeconomic groups by national statistics [11,34] may not be attributed to a reduced acceptability of these foods compared to children from higher socioeconomic segments. This is an encouraging insight, as it suggests that the food environment associated with socioeconomic status may not negatively influence the acceptance of the familiar vegetables analyzed. However, other factors may be influencing the lower vegetable consumption in vulnerable groups, as sex and BMI showed only a limited impact on vegetable liking in low socioeconomic groups. Specifically, an interaction effect was observed in males, where overweight or obese boys rated vegetables higher than those with regular weight. On the other hand, a significant interaction between SES and BMI was found only in children from high socioeconomic backgrounds, where overweight or obese children rated vegetables higher than their normal-weight counterparts. Additionally, children from higher socioeconomic backgrounds were more stringent in their sensory evaluation of aroma and appearance, yet this did not translate into differences in overall liking scores. Interestingly, familiar vegetables that were less liked when cooked received higher ratings when presented raw, emphasizing the impact of preparation strategies on children’s acceptance. Notably, this effect persisted even when the raw presentation was unfamiliar, suggesting that preparation methods can override initial reluctance and enhance acceptability. These findings highlight the importance of considering both availability and preparation methods as key factors in promoting vegetable consumption among children. Addressing structural barriers in food environments and implementing simple preparation strategies could offer practical solutions to improve dietary quality, particularly in lower socioeconomic groups. These insights could support public health initiatives aimed at improving vegetable intake among children, while future research should explore policy-driven strategies to enhance vegetable acceptance in children by focusing on their sensory properties across socioeconomic contexts.

## Figures and Tables

**Table 1:** Samples tasted in the first research stage and their preparation.

Sample	Preparation	Serving Temperature
Tomatoes	Raw and slightly seasoned with salt and sunflower oil	Room temperature
Lettuce	Raw and slightly seasoned with salt, lemon, and sunflower oil	Room temperature
Corn	Boiled with salt and slightly seasoned with sunflower oil	Room temperature
Cucumber	Raw and slightly seasoned with salt, lemon, and sunflower oil	Room temperature
Carrots	Boiled with salt	Warm
Beets	Boiled and slightly seasoned with salt, lemon, and sunflower oil	Room temperature
Broccoli	Boiled with salt	Warm
Cauliflower	Boiled with salt	Warm

**Table 2:** Demographic characterization of participating children in the first research stage (*n* = 363).

Variable	Category	*n*	%
SES *	Low	65	17.9
	Low-medium	82	22.6
	Medium	66	18.2
	Medium-high	77	21.2
	High	73	20.1
Sex	F	193	53.2
	M	170	46.8
BMI	Overweight–obesity	178	49.0
	Normal weight	185	51.0

* SES: Socioeconomic status; sex: children’s sex; BMI: body mass index category. Distribution of BMI within each SES group: low SES (53.8% overweight–obesity and 46.2% normal weight), low-medium (54.9% overweight–obesity and 45.1% normal weight), medium SES (54.5% overweight–obesity and 45.5% normal weight), medium-high (67.5% overweight–obesity and 32.5% normal weight), and high SES (23.3% overweight–obesity and 76.7% normal weight).

**Table 3:** Samples tasted in the second research stage and their preparation.

Sample	Preparation	Serving Temperature
Carrots Raw	Raw	Room temperature
Carrots Cooked	Boiled with salt	Warm
Cauliflower Raw	Raw and slightly seasoned with salt, lemon, and sunflower oil	Room temperature
Cauliflower Cooked	Boiled with salt	Warm
Beets Raw	Raw and slightly seasoned with salt, lemon, and sunflower oil	Room temperature
Beets Cooked	Boiled and slightly seasoned with salt, lemon, and sunflower oil	Room temperature

**Table 4:** Demographic and behavioral characterization of all participating children in the second research stage (*n* = 191) *.

Variable	Category	*n*	%
SES	Low	58	30.4
	Medium	68	35.6
	High	65	34.0
Sex	F	108	56.5
	M	83	43.5
BMI	Overweight–obesity	92	48.2
	Normal weight	99	51.8

* SES: Socioeconomic status; sex: children’s sex; BMI: body mass index category. Distribution of BMI within each SES group: low SES (53.4% overweight–obesity and 46.6% normal weight), medium SES (67.6% overweight–obesity and 32.4% normal weight), and high SES (23.1% overweight–obesity and 76.9% normal weight).

## Data Availability

The data presented in this study are available on request from the corresponding author. The data are not publicly available due to privacy restrictions.
